# Assessment of Inoculation Methods of *Thielaviopsis paradoxa* (De Seynes) Höhn into Oil Palm Seedlings under Greenhouse Conditions

**DOI:** 10.3390/jof7110910

**Published:** 2021-10-27

**Authors:** Sandra Gaitán-Chaparro, Edwin Navia-Rodríguez, Hernán Mauricio Romero

**Affiliations:** 1 Colombian Oil Palm Research Center—Cenipalma, Oil Palm Biology and Breeding Research Program, Bogotá 11121, Colombia; slgaitanc@unal.edu.co (S.G.-C.); enavia@cenipalma.org (E.N.-R.); 2 Department of Biology, Universidad Nacional de Colombia, Bogotá 11132, Colombia

**Keywords:** spear leaf rot, inoculation methods, interspecific oil palm hybrid, plant–pathogen interactions, *Thielaviopsis paradoxa*

## Abstract

Oil palm (*Elaeis guineensis* Jacq. and *Elaeis Oleifera* Cortes) is one of the most important oil crops in the world. Colombia is the fourth-largest oil palm producer worldwide. However, oil palm diseases are a significant factor affecting yield. *Thielaviopsis paradoxa* (De Seynes) Höhn is a pathogen that affects young palm trees, causing spear rot. Four disease establishment methods were studied to replicate, in a controlled environment, the symptoms of the disease found in the field. Young palm trees were inoculated with a suspension of endoconidia using either local infiltration, drip, scissor cut, or direct contact with agar blocks bearing mycelia and conidia. The effects of the inoculation methods were studied in dose-method-disease severity experiments conducted in a greenhouse under controlled conditions. All four methods resulted in *T. paradoxa* infections and the development of symptoms of the disease. The disease severity was correlated with the method and dose of inoculation. In trials to test Koch’s postulates, *T. paradoxa* was isolated from areas of disease progression in the inoculated trees, but the teleomorph *Ceratocystis paradoxa* (Dade) Moreau was not observed. A photographic record of the infection process at different times post-infection was compiled. Given that establishing the disease through artificial inoculation is essential for assessing plant pathogenesis, this study determined that the local infiltration method (1 × 10^6^ endoconidia mL^−1^) and a 3–7 day incubation period were critical for the development of symptoms as severe as those observed in natural infections in the field.

## 1. Introduction

The genus *Elaeis* belongs to the monocotyledonous family Arecaceae. It comprises two taxonomically well-defined species, the African oil palm *Elaeis guineensis* and the American oil palm *Elaeis oleifera*. Oil palm is one of the most important oil crops globally, given that more than 33% of oils and fats consumed worldwide are derived from it. Moreover, oil palm is the most productive oil crop, yielding approximately 4 tons oil ha^−1^ year^−1^, a figure nearly ten times higher than the yields of other important oil crops [1]. In South America (Ecuador, Brazil, Peru, Venezuela) and Central America (Costa Rica), the primary barrier to oil palm crop development is the plant’s susceptibility to pests and pathogens, the control of which can account for up to 30% of production costs [2,3].

One of the pathogens of oil palm is *Thielaviopsis paradoxa*, the anamorph of *Ceratocystis paradoxa,* which belongs to the family Ceratocystidaceae [4]. It is a pathogen with a broad host range, including economically important crops, such as sugarcane, date palm, banana, sorghum, cocoa, sweet potato, coconut, pineapple, corn, and other palm species [5,6,7]. *T. paradoxa* can infect any part of the plant, creating a wide variety of symptoms. Diseases caused by *T. paradoxa* have a high destructive potential for oil palm plantations. They have been designated in many different ways in the literature (stem exudation, black burn, spear leaf rot, and bud rot) [4,5]. These names describe symptoms that may or may not be expressed in each instance and reflect a particular symptomatology in specific palm taxa. In Italy, this fungus has been reported as a causal agent of stem rot in *Phoenix dactylifera* [6,7] and *Howeia forsteriana* [8]. The fungus has also been reported as the causal agent of bunch discoloration disorder in most date palm-producing countries, including Saudi Arabia [9,10,11], Iraq, Kuwait [12], Qatar [13], and Egypt [14]. Additionally, this fungus has been reported as the causal agent of bud rot in *Hyophorbe lagenicaulis* (bottle palm tree) in Thailand [15], as well as in other palm species, such as *Areca catechu*, *Hyophorbe lagenicaulis, Phoenix africanus*, *Rhapis* sp., *Roystonea elata*, *Sabal palmetto*, *Syagus romanzoffiana,* and *Washingtonia filifera* [4,16,17]). Additionally, the pathogen has been described as the causal agent of basal stem rot in ornamental oil palms in China [18]. *T. paradoxa* is considered an aggressive and difficult-to-control pathogen that can be dispersed by rain, wind, wounds, and the tools used to harvest or eradicate diseased plants [5,7,19]. Several studies have been conducted to control this pathogen with biological and chemical methods in different hosts [6,17,18]

In Colombia, *T. paradoxa* has been associated with both spear leaf (youngest leaf) rot and bud rot. In spear leaf rot, this pathogen results in a significant disease in the neotropics that affects young trees. The initial stage is characterized by the onset of small necrotic areas on the spear leaf that extend throughout the leaf until it collapses. In some cases, this rot reaches the meristem, causing the death of the palm. In other cases, the palm controls the infection. As a result, the palm begins a recovery process, with the emission of small and deformed spear leaves at the beginning, until reaching the emission of normal spear leaves [19]. In bud rot, a disease of the oil palm caused by *Phytophthora palmivora* that destroys the terminal bud and adjacent leaves resulting in tree death, *T. paradoxa* behaves like an opportunistic agent [3]. In some regions of America, the planting of African oil palm cultivars (*E. guineensis*) is limited by the bud rot complex.

For this reason, oil palm breeders became interested in the American oil palm (*E. oleifera*) because it is a source of many economically valuable traits, such as resistance to several diseases, slow height increment, and improved oil nutritional value [20]. Thus, interspecific O × G (*E. oleifera* × *E. guineensis*) hybrids were developed. However, to date, the interaction O × G hybrids-*T. paradoxa* has not been characterized.

The objective of this work was to assess four methods for the inoculation of oil palm with *T. paradoxa* under controlled conditions to set up a *T. paradoxa*-oil palm pathosystem that may be used in omics studies. First, we evaluated de inoculation methods on *E. guineensis* and then on O × G hybrids. The selected methods have been described in the literature for *Thielaviposis basicola* and *Alternaria brassicae* on detached leaves and adapted to greenhouse conditions [21,22].

The development of these methods will allow researchers to conduct pathogenesis studies of *T. paradoxa* in *E. guineensis* and the interspecific O × G hybrids, which would lead to the characterization of the infection process and the evaluation of the susceptibility of oil palm genotypes during their interaction with the fungus.

## 2. Materials and Methods

### 2.1. Plant Materials

Two types of cultivars were considered for this experiment: (i) Six-month-old *E. guineensis (tenera)* nursery plants and (ii) four-month-old O × G hybrid nursery plants. The *E. guineensis* cultivars were used to evaluate *T. paradoxa* inoculation methods in a susceptible material. The second type corresponded to hybrids selected as promissory. These hybrids showed excellent agronomic and productive performance and good behavior against diseases and pests. All the plants were kept in polyethylene bags, 15 cm in diameter and 18 cm in height, and placed in a greenhouse at the Unipalma plantation in Cumaral, Meta (geographic coordinates are latitude: 4.271°, longitude: −73.487°, and elevation: 412 m) during the whole experiment. The bags were filled with a mix of soil: vermiculite 3:1. The region has an average annual rainfall of 2800 mm, with a relative humidity of 80%, a luminosity of 1482 h, an average temperature of 27 °C, a minimum of 20 °C, and a maximum of 32 °C.

### 2.2. Fungal Inoculum

The fungal isolate used in this study belongs to a collection of lyophilized strains of *T. paradoxa* collected in different regions of Colombia from *E. guineensis* palms with varying degrees of severity of spear leaf rot. During previous studies, the strains were identified at the molecular level (sequences of the internal transcribed spacer regions (ITS) were used) [23]. Their pathogenicity was evaluated on oil palm leaf fragments using 0.4% agar with *T. paradoxa* mycelium and establishing the total percentage of tissue invaded for 15 days. With the results, the strains were categorized according to aggressiveness (low < 40%, intermediate 40–60%, and high > 60% of invaded tissue). Aggressiveness was expressed as the percentage of external and internal tissues invaded by the fungus at the end of the observation time. Differences among strains were analyzed using the Tukey test, and the strain producing the highest disease severity was selected for the present study.

Four inoculation methods were assessed using two inoculum types. The inocula were prepared using monosporic isolates: (i) agar block and (ii) spore suspension (endoconidia). After obtaining *T. paradoxa* mycelium on potato dextrose agar (PDA) and growing it at ambient temperature (22 °C to 24 °C), blocks of agar (0.5 cm square) were cut from the periphery of a 7-day culture. The endoconidial suspension was prepared by adding 10 mL of Tween 80 (0.03% in distilled water) to the sporulated culture (10 days), and endoconidia were collected using fiberglass filtration; three concentrations of 1 × 10^4^, 1 × 10^5,^ and 1 × 10^6^ endoconidia/mL were established using Neubauer chamber count.

### 2.3. Inoculation Methodology

Four methods were used to inoculate seedlings with *T. paradoxa*: drip, infiltration, cutting, and direct contact with cultured agar blocks. The seedlings were maintained for 15 days under control conditions, monitoring temperature and humidity using a data logger thermohygrometer. Humidity was held at 80–100%, and the temperature was maintained at 26–28 °C, from the moment of the inoculation. (i) Inoculation with Mycelium blocks: agar blocks containing mycelium were placed at the base of the third leaf, which had previously been given a 2 mm diameter round surface wound with a pipette tip. The agar blocks were covered with parafilm to guarantee humid conditions. Sterile agar blocks were used as controls. (ii) Local Infiltration: a hypodermic syringe was used for infiltrating 0.1 milliliters of endoconidial suspension at the base of the third leaf. The infiltration buffer was used as the control. (iii) Cutting of the Leaf: the apex of the third leaf was cut using scissors previously soaked in the endoconidial suspension. For control, scissors were soaked in a buffer solution. (iv) Drip Inoculation: one milliliter of endoconidial suspension was drip-applied at the base of the third leaf, which had previously been given a superficial wound. A buffer drip was used as a control.

In the dose-response trial, the disease was assessed 15 days after fungal inoculation at 10^4^, 10^5^, and 10^6^ endoconidia mL^−1^. Each seedling was considered a replicate, with three replicates per treatment. The complete experiment was repeated three times.

In the time-disease trial, symptom development was assessed at 0, 24, 48, 72, 96, 120, 240, and 360 h post-infection (hpi).

### 2.4. Disease Severity

The lesioned area on the leaf was measured at each post-inoculation assessment time point. The severity of the disease in each of the thirty O × G palms was recorded. The disease severity was estimated using the Chiang et al. [24] formula (Equation (1)):

Disease Severity = Mean area of the affected tissue/Mean of the total spear-leaf area × 100(1)

Severity grades were assigned according to the following scores:

0 = No lesion;

1 = Presence of wet lesions;

2 = Presence of necrotic lesions;

3 = Leaf necrosis of less than 50%;

4 = Leaf necrosis of more than 50%;

5 = Necrosis of a large portion of the leaf, leading to the death of the leaf.

The effect of each treatment on the disease severity or the dose-time relationship was analyzed by one-way analysis of variance (ANOVA) using the XLSTAT software (version 2015.5, Addinsoft Inc., New York, NY, USA). Differences between methods were analyzed using Fisher’s least significant difference test.

### 2.5. Assessment of the Infection Process

O × G hybrids were used to evaluate the four inoculation methods and the infection process at the microscopic scale. Altogether, ten post-inoculation times were assessed (6, 12, 18, 24, 48, 72, 96, 120, 240, and 360 hpi), and three replicates were included each time. The whole experiment was repeated three times. The leaf tissue was treated with 10% KOH at 90 °C for 15 min to remove the cytoplasmic content while preserving cell walls, enhancing the ability to view fungal structures under the microscope using ink staining. The sampling of the leaf tissue was destructive.

Additionally, samples from the lesion progression area (area with diseased and healthy tissue in proportion 30–70) were taken to prove Koch’s postulates. The small tissue cuts (1–2 cm long) were disinfected with 10% sodium hypochlorite for 1 min, followed by washing with sterile distilled water, and transferred to an isolation medium (PDA) and incubated at room temperature (22 °C to 24 °C).

## 3. Results

### 3.1. Thielaviopsis sp. Isolation

**Figure 1:**
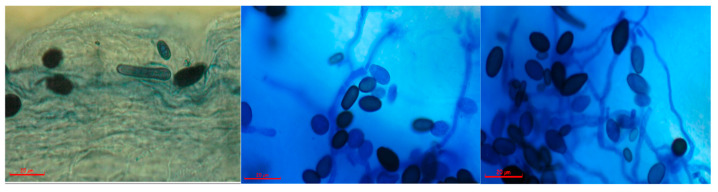
Microscopic characteristics of Thielaviopsis sp. isolated from infected tissue. The photographs show septate hyaline hyphae, microconidia, chlamydospores, and conidiophores. The red bar corresponds to 20 μm.

The fungus *Thielaviopsis* sp. was isolated from the leaf of symptomatic nursery O × G hybrids and was morphologically characterized. *T. paradoxa* forms thick-wall spores and chlamydospores that produce infectious asexual spores or conidia. Two types of conidia were observed in fresh preparations from culture and infected tissue; the first was small, cylindrical, hyaline to pale brown microconidia, 6.3–10.7 × 2.3–4.1 μm in size. The second type was notably larger (11.8–16.2 × 5.9–8.6 μm), brown in color, oval (Chlamydospores). They were responsible for the black color in the final stage of the disease development. The conidiophores observed were straight, hyaline-colored, with conidia forming in chains (Figure 1). The morphological characteristics coincide with the previous descriptions of *Thielaviopsis paradoxa* (De Seynes) Höhn [21].

### 3.2. Comparison of Inoculation Methods

Inoculations of *E. guineensis* seedlings with *T. paradoxa* were successful. The symptoms were developed with all four inoculation methods in plants kept for 15 days under controlled conditions. Although all *E. guineensis* seedlings showed progressive symptoms of the disease, the morphology of the lesions varied in accordance with the inoculation method used. In plants inoculated using cutting and mycelium block, necrosis began at the injury site and advanced slowly towards the leaflets. With local infiltration and drip of the endoconidial suspension, wet necrotic lesions developed around the inoculation site and advanced through the petiole towards the spear leaf. Disease symptoms caused by infiltration and drip were similar to those observed in the field and described in other studies [15,19].

**Figure 2:**
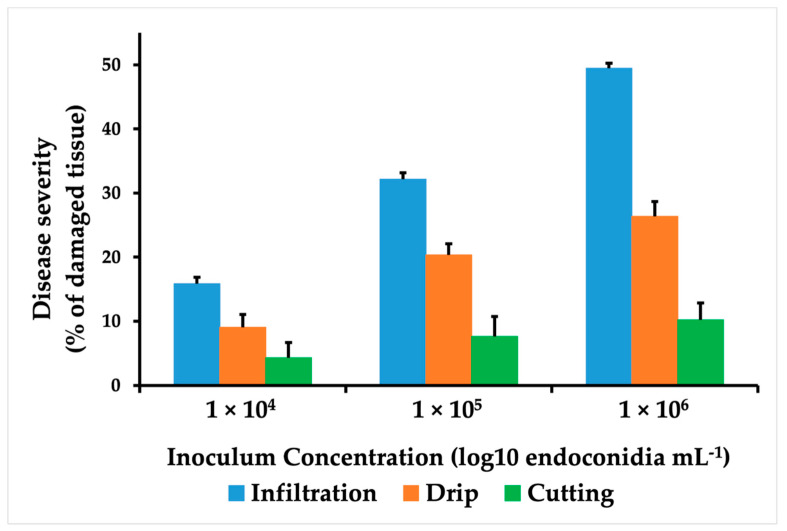
Inoculation method comparison. *E. guineesis* disease severity in seedlings inoculated with different concentrations of *T. paradoxa* spores (1 × 10^4^, 1 × 10^5,^ and 1 × 10^6^ endoconidia per milliliter). The methods used were infiltration (blue), cutting with scissors (green), and drip (orange). Values correspond to the means of nine replications at 240 hpi. Error bars correspond to mean standard error.

The onset and lesion progression over time depended on inoculum dose and the inoculation method. Infiltration resulted in the shortest incubation period (48 h) and the fastest rate of lesion progression. The effect of inoculum concentration on disease severity was studied for each inoculation method, with lesion progression only being detected at concentrations of 10^5^ and 10^6^ (Figure 2). Variance analysis for the severity and assessment time interaction showed a significant difference for the infiltration method (*p* = 0.003). Statistical differences were significant for the concentration of the inoculum used in the injection and drip methods (*p* = 0.020), leading to the continuation of the experiments without using the 1 × 10^4^ concentration.

**Figure 3:**
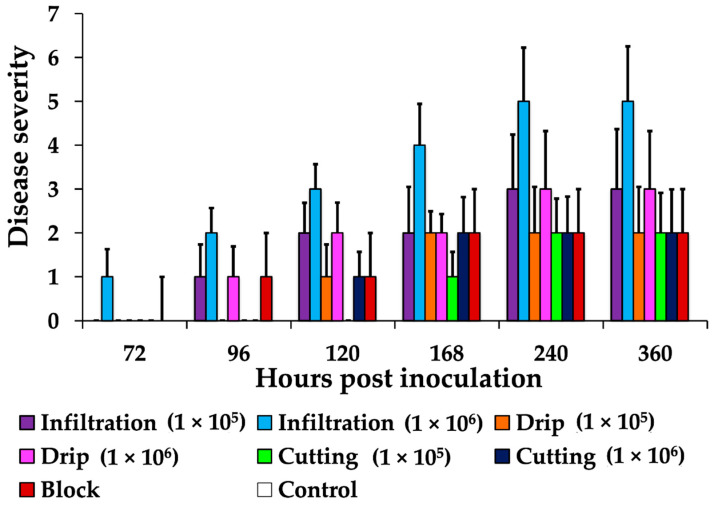
Disease severity in O × G hybrid seedlings inoculated with *T. paradoxa* suspensions (1 × 10^5^ and 1 × 10^6^ endoconidia mL^−1^) using local infiltration, scissor cutting, drip, and agar blocks. In all, nine replicates were included for each time. No increased disease severity was observed after 240 hpi with any of the methods.

Interspecific O × G hybrid seedlings were inoculated by the previously evaluated methods in *E. guineensis.* Seedlings were observed for initial lesions every 24 h. The progression of lesions was monitored over two weeks and classified in accordance with the degree of severity of the disease. The development of a small necrotic area surrounded by a yellow ring was observed at 72 h in the spear leaf base of seedlings inoculated using the infiltration approach (1 × 10^6^). At 96 hpi, seedlings inoculated using agar block and drip methods showed the same type of initial lesion. With the scissor cut method, lesions were observed only after 96 h (Figure 3).

Necrotic lesions ascending from the base towards the end of the leaf were observed at 120 hpi for the infiltration method (with the two inoculum concentrations) and the drip method (with the 1 × 10^6^ concentration only). Lesions in the spear leaf were present at 240 hpi. The leaves showed varying degrees of severity, with necrosis and spear leaf collapse evident only with the injection of inoculum at a concentration of 1 × 10^6^. The final observation was made 15 days following inoculation, at which time there was no evidence of further lesion progression in any of the methods under assessment (Figure 2).

Tukey’s multiple comparison test with a 95% confidence level in the interaction method–inoculum concentration showed that the highest percentages of damage occurred with the infiltration method (1 × 10^6^ endoconidia mL^−1^), which was statistically different from the rest of the treatments (B). With this method, more than 50% affection was observed in the spear leaf. A second group (AB) consisted of the infiltration methods with 1 × 10^5^ endoconidia mL^−1^ and the dripping at the base of the spear leaves with 1 × 10^6^ endoconidia mL^−1^ (Table 1).

The analysis of variance determined that the inoculation method that allowed the reproduction of the symptoms observed in the field and all the stages described in the severity scale was infiltration with an inoculum concentration of 1 × 10^6^ endoconidia mL^−1^. Although it can be considered a severe method of inoculation due to the damage caused by the needle, no symptoms or lesions like those caused by *T. paradoxa* were evident in any of the controls.

### 3.3. Infection Characterization

**Figure 4:**
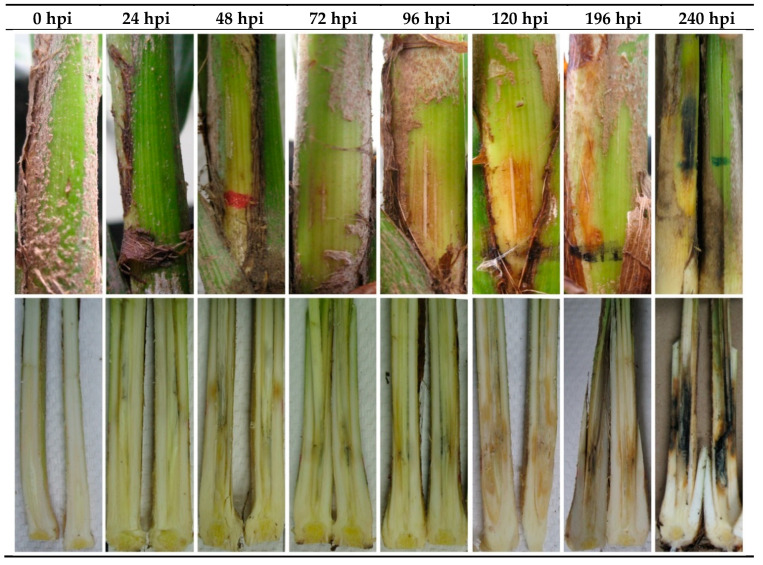
Disease progression in oil palm hybrid O × G seedlings inoculated using local infiltration of a *T. paradoxa* suspension of 1 × 10^6^ endoconidia mL^−1^. The externally and internally. The external damage (upper panel) and internal damage (bottom panel) were recorded at different hours post-infection (hpi).

Variance analyses determined that the inoculation method that resulted in symptoms as severe as those observed in the field was infiltration using an inoculum concentration of 1 × 10^6^ endoconidia mL^−1^. The first external evidence of infection was a yellow ring at the inoculation site, which was observed at 72 hpi; the lesion area increased considerably at 96 hpi. The ring extended along the spear leaf, with a necrotic tissue appearance and spear leaf collapse at 240 hpi time. However, the results of destructive sampling showed that lesions were first apparent at 24 hpi, beginning with rot and advancing rapidly along the immature tissues of the petiole. By 120 h, more than 50% of the petiole was affected with rot lesions with a fermented smell, resulting in spear leaf collapse at approximately 240 h. As shown in Figure 4, the external damage caused by the fungus (upper panel) does not reflect the internal damage during infection (bottom panel).

**Figure 5:**
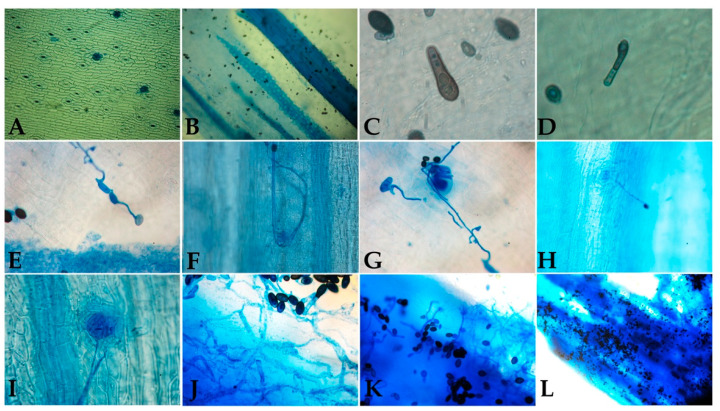
Infection process of O × G oil palm hybrid seedlings after *T. paradoxa* inoculation with a 1 × 10^6^ endoconidia mL^−1^ suspension using the local infiltration method. (**A**) Healthy oil palm petiole tissue (10× magnification). (**B**) Six hpi (10× magnification). (**C**) Germ tube formation 12 hpi (100× magnification). (**D**) Septum formation and hyphae colonization 18 hpi (100× magnification). (**E**) Hyphae connections 24 hpi. (**F**,**G**) Hyphae around the stomata (100× magnification). (**H**,**I**) Germinated endoconidium with hyphae advancing towards the stoma (10× and 100× magnification, respectively). (**J**) Tissue colonization 36 hpi (40× magnification). (**K**,**L**) Sporulation process between 76 hpi and 96 hpi (40× and 10× magnification, respectively). hpi = hours post-inoculation.

Following physical contact between *T. paradoxa* and palm tissue, the infection was initiated by germination of endoconidia. Germ tubes were first evident after 12 hpi, and hyphae formation was observed at approximately 18 h. On occasion, these hyphae connected with hyphae from other endoconidia and extended towards the stomata. At 72 hpi, branching of hyphae within the tissue was first observed, followed by the presence of endoconidia throughout the necrotic tissue at 96 hpi (Figure 5).

## 4. Discussion

In this work, four inoculation methods were adapted and evaluated to infect oil palm spear leaves with *T. paradoxa*. Lesion progression and symptoms in complete plants under greenhouse conditions were recorded as a requirement for establishing a *T. paradoxa*-oil palm pathosystem. Complete plants were preferred because, although detached parts of plants are the most suitable plant material to inoculate pathogens at a confined level [22], they could show a series of responses related to the reaction against infection by pathogens and general stress and senescence [25]. Furthermore, key signaling pathways could be lost because of the lack of connection between roots and stem [26].

The inoculation method with the best results was local infiltration with a 1 × 10^6^ conidia per ml suspension. It was the most appropriate method because the area of injury was minimal and, more importantly, it allowed reproducing all the symptoms observed in the field. All four inoculation methods produced infection with *T. paradoxa* and induced the disease symptoms. However, there were differences in disease progression and severity depending on the inoculation method. Furthermore, the results obtained with the infiltration and drip methods were consistent with those reported by studies of inoculation of *T. paradoxa* in whole plants [14,27].

The results with agar disc and cutting methods were different from other studies. The agar disc method was modified because it was considered a very aggressive method, making a punch wound of 5 mm in diameter and 5 mm deep to inoculate the PDA disc with mycelium and endoconidia, as described previously [14,15,27]. The cutting method with scissors impregnated with an endoconidia suspension had the drawback of causing considerable variation in spore distribution, precluding the determination of a mean inoculum concentration and volume, in addition to producing an injury of significant size. Although, in most methods, lesions are used in artificial inoculations, they should be mild because if a response is being evaluated at the molecular level, it will not be clear whether that response is due to the invasion of the fungus or the intensity of the wound.

Block and cut inoculation methods produced lower severity levels of disease, possibly because the inoculum was less, and plant defense response is rapidly induced in tissues with large injured areas [22]. Although disease progression with the drip method was initially rapid, the final severity levels were low, and lesions remained confined to less than 25% of the tissue. The local infiltration method appears to be the most adequate considering that the volume of the inoculated pathogen suspension is known and the injury area is small.

The disease establishment and symptom manifestations were correlated with pathogen inoculum. The higher the inoculum concentration, the higher the infection. As the inoculum concentration increased, the disease symptoms appeared rapidly. On the contrary, the disease developed slowly at low inoculum concentrations, and the symptoms appeared late with no evidence of progression. This correlation between disease severity and inoculum concentration has been observed in studies of *T. basicola* [28]. The external symptoms were evident at 72 hpi, with a slight yellow halo at the inoculation site. The symptoms spread along the spear leaf, with the appearance of necrotic tissue and the collapse of the spear leaf at 240 hpi. However, inside the seedlings, the affected tissue was observed at 24 hpi.

The characterization of the infection process allowed the establishment of the time it took to develop the pathogen structures that invaded the host tissue; 12 h for conidia germination, 24 h for hyphae formation, 48 h for hyphae growth and interconnection, 72 h for tissue invasion, and 96 h for endoconidia formation. These results are of great interest in omics studies to determine the sampling times according to the focus of the research. The observation of interconnected hyphae near to stomata suggests that, like other phytopathogens, *T. paradoxa* penetrates through natural openings such as stomata [14,29]. Different ways of entry include mechanical wounds or those caused by insects that feed on the palm, such as *Rhynchophorus palmarum*, which has been postulated as a probable vector of the fungus. This hypothesis is reinforced because *T. paradoxa* is present in the insect’s digestive tract [30].

The seedling tissue cuts showed that an exogenous signal was required for *T. paradoxa* germ tube development to initiate, which occurred at approximately 12 hpi. This period was more extended than that observed for *T. basícola* in tobacco root, which was reported to be 8 h [26].

## 5. Conclusions

Establishing a disease using artificial inoculations is essential for pathogenesis studies in plants. In our research, local infiltration with an inoculum concentration of 1 × 10^6^ endoconidia mL^−1^ and an incubation period of 3 to 7 days were determinant factors for seedlings to develop symptoms as severe as those observed in the field. Establishing this method allowed the observation of the development stages of the disease, which supported the definition of a severity scale. Establishing the disease by artificial inoculation was essential to determine an infection process timeline, the first step for sampling in RNASeq studies during plant-pathogen interaction. Furthermore, the infection process timeline could be used to study etiology, disease resistance, and disease control. Finally, the infiltration method could be used in other species of the Arecaceae family affected by *T. paradoxa*, adjusting the inoculum concentration.

## Figures and Tables

**Table 1:** Summary of pairwise comparisons for different inoculation methods.

(Tukey’s Honest Significant Difference Test)			
Inoculation Method	Least Squares Means	Groups	
Cutting 1 × 10^5^	2.500	A	
Agar Block	3.000	A	
Cutting 1 × 10^6^	3.000	A	
Drip 1 × 10^5^	3.167	A	
Drip 1 × 10^6^	4.667	A	B
Infiltration 1 × 10^5^	6.167	A	B
Infiltration 1 × 10^6^	11.167		B

## Data Availability

The data presented in this study are available on request from the corresponding author. The data are not publicly available due to privacy restrictions.
